# Undergraduate Medical Science Students Positive Attitude towards Online Classes during COVID-l9 Pandemic in a Medical College: A Descriptive Cross-sectional Study

**DOI:** 10.31729/jnma.5413

**Published:** 2021-02-28

**Authors:** Gita Dhakal Chalise, Mamata Bharati, Jayendra Bajracharya, Ambu KC, Subhadra Pradhan, Bibhav Adhikari, Manoranjan Shrestha

**Affiliations:** 1 College of Nursing, Nepalese Army Institute of Health Sciences, Kathmandu, Nepal; 2 Department of Biochemistry, College of Medicine, Nepalese Army Institute of Health Sciences, Kathmandu, Nepal; 3 Little Angels College of Management, Kathmandu, Nepal

**Keywords:** *COVID-19*, *lockdown*, *MBBS*, *nursing*, *online classes*

## Abstract

**Introduction:**

The outbreak of COVID-19 led to lockdown, which in turn led to the closure of schools and colleges. This situation created an opportunity to transform the conventional learning methods into an online or virtual method using various digital platforms. Nepalese Army Institute of Health Sciences started online classes as an alternative way to resume education during this pandemic. Therefore, this study aims to identify the prevalence of medical science students with a positive attitude towards online classes during the COVID-19 pandemic in a medical college of Kathmandu, Nepal.

**Methods:**

The study was conducted among 513 students using descriptive cross-sectional study design who were currently studying Bachelor of Medicine, Bachelor of Surgery, Proficiency Certificate Level Nursing, Bachelor of Science in Nursing, and Bachelor of Nursing Science under the Nepalese Army Institute of Health Sciences. Data was collected from June-July 2020 through an online self-administered questionnaire using Google forms. The results were presented through frequency, percentage, mean, and standard deviation.

**Results:**

In this study, 112 (87.5%) Bachelor of Science in Nursing, 189 (83.6%) Bachelor of Medicine, Bachelor of Surgery, and 63 (82.9%) Bachelor of Science in Nursing students had a positive attitude towards online classes, while 51 (61.5%) of Proficiency Certificate Level Nursing students had a negative attitude towards it.

**Conclusions:**

Most bachelor-level students had a positive attitude towards online classes. With a positive attitude, students' participation and adaptability in online classes will be high, resulting in better academic performance.

## INTRODUCTION

Nepal had imposed a phased lockdown to prevent the spread of COVID-19, resulting in schools and universities remaining closed. This necessitated the provision of online learning for strengthening students' learning skills and sustaining their motivation. Online learning was initiated at the Nepalese Army Institute of Health Sciences on 22nd April 2020. Tribhuvan University had approved online classes to their constituent and affiliated colleges and issued guidelines on 25th April.^1-5^

Due to the intense curriculum, medical and nursing students have to adhere to their classroom lectures and bedside practicum simultaneously. Frequent rotations in these areas make them potential vectors for COVID-19. Therefore, the students' physical and mental safety should be prioritized by taking appropriate measures, including online learning implementation.^6-9^ Online learning refers to the method of content dissemination and rapid learning through the application of information technology and internet technology using various online platforms.^10,11^

The study aimed to identify the positive attitude of students towards online classes during the COVID-19 pandemic.

## METHODS

A descriptive cross-sectional study had been conducted among nursing and MBBS students from the Nepalese Army Institute of Health Sciences (NAIHS), affiliated with Tribhuvan University (TU). The study included the proficiency certificate level (PCL) and bachelor level nursing students from the College of Nursing and MBBS students from the College of Medicine. Data were collected from June to July 2020.

Ethical approval for the study had been taken from the Institutional Review Committee (IRC) of NAIHS (Regd. No. 294/May 2020). Written permission for the study was also taken from the administration of NAIHS, College of Medicine and College of Nursing. Informed written consent was obtained from each student which was attached in Google form. Anonymity was maintained by asking students not to write their names on questionnaires. The sample size was calculated as follows;


$$\begin{array}{ll} \text{n} & ={\text{Z}}^{\text{2}}\times \text{p}\times \text{q}/{\text{e}}^{\text{2}}\\ & ={\left(1.96\right)}^{2}\times 0.5\times (1-0.5)/{\left(0.05\right)}^{\text{2}}\\ & =384 \end{array}$$

Where,

Z = 1.96 for confidence interval at 95%
p = prevalence 50% for maximum sample size
q = 1-p
e = margin of error 5%

The minimum sample size was calculated to be 384. Adding 20% of the non-response rate for missing and incomplete data estimated minimum sample size was 461.

There were altogether 922 students currently studying in MBBS, PCL nursing, Bachelor of Science in Nursing (BSN), and Bachelor of Nursing Science (BNS) stream under NAIHS. So, for finite population adjustment,

n'= n/[1+{(n'1)/N}]

Here n is Cochran's sample size recommendation, N is the population size, and n' is the new adjusted sample size.


$$\begin{array}{l} \text{= 384.6}/\text{[1}+\text{(384.6}-\text{1)}/\text{472)]}\\ \text{= 212} \end{array}$$

Semi-structured self-administered questionnaires had been used for data collection. These questionnaires were developed in Google form containing two sections: the first section on socio-demographic information and the second section on students' attitudes towards online classes. Attitude had been measured by using a 5-point Likert Scale, i.e., strongly disagree, disagree, neutral, agree, and strongly agree. These were summed into positive and negative attitudes by Statistical Package for Social Science (SPSS) software during analysis. Questions were developed after reviewing relevant papers on a related topic.

Data collection was done by using an online self-administered technique of Google form. The Google form link had been posted in the Google classroom and requested to be filled within two weeks and sent back to the researcher. After two weeks, follow-up was done with students by reposting in Google classroom and requested to submit within one week by remaining students. Participants had been provided instruction about the proper way to fill up the questionnaire. Data analysis was done using a statistical package for social science (SPSS version 16) software and described using descriptive statistical techniques, i.e., frequency, percentage, mean and standard deviation.

## RESULTS

The study included 513 students, among which 287 (55.9%) were nursing, and 226 (44.1%) were MBBS. Among nursing students, 128 (44.6%) were BSN, 83 (28.9%) were PCL nursing, and 76 (26.5%) were BNS. The mean age of student was: PCL nursing 17.86 years (SD±1.191), BSN 20.29 years (SD±1.603), MBBS 21.24 years (SD±1.915), and BNS 24.80 years (SD±2.572). In nursing, all 287 (100%) students were female, whereas, in MBBS, 144 (63.7%) were male, and 82 (36.3%) were female.

The majority of students (72.3% to 86.7%) from all streams used WiFi for their online classes (Table 1).

**Table 1:** Age, gender, sources of internet and internet cost per month.

Variables	PCL Nursing n (%)	BNS n (%)	BSN n (%)	MBBS n (%)
Age (in years)^*^				
≤20	82 (98.8)	1 (1.3)	70 (54.7)	85 (37.6)
21-25	1 (1.2)	45 (59.2)	57 (44.5)	139 (61.5)
≥26	-	30 (39.5)	1 (0.8)	2 (0.9)
Total	83 (100)	76 (100)	128 (100)	226 (100)
Gender				
Female	83 (100)	76 (100)	128 (100)	82 (36.3)
Male	-	-	-	144 (63.7)
Total	83 (100)	76 (100)	128 (100)	126 (100)
Mode of internet connection				
Cellular data	23 (27.7)	12 (15.8)	17 (13.3)	51 (22.6)
WiFi	60 (72.3)	64 (84.2)	111 (86.7)	175 (77.4)
Total	83 (100)	76 (100)	128 (100)	226 (100)
Internet cost per month (in NPR)^#^				
≤1000	27 (32.5)	13 (17.1)	29 (22.7)	68 (30.1)
1001 - 1500	19 (22.9)	26 (34.2)	51 (39.8)	71 (31.4)
1501-2000	35 (42.2)	34 (44.7)	43 (33.6)	73 (32.3)
>2000	2 (2.4)	3 (3.9)	5 (3.9)	14 (6.2)
Total	83 (100)	76 (100)	128 (100)	226 (100)

* Mean age of students: PCL nursing (mean ± SD, 17.86, ±1.191), BNS (mean ± SD, 24.80 ± 2.572), BSN (mean ± SD, 20.29 ±1.603), MBBS (mean ± SD, 21.24 ± 1.915)

# Mean internet cost: PCL nursing (mean ± SD,1385.12 ± 511.392), BNS (mean ± SD, 1580.66 ± 575.753, BSN (mean ± SD,1420.86 ± 493.424), MBBS (mean ± SD,1449.90 ± 638.981)

Regarding students' attitude towards online classes, 353 (68.8%) students agreed and strongly agreed on slow computers and poor internet connections discourage them to participate in the online classes; 345 (67.3%) preferred the traditional face-to-face mode of classroom teaching than online classes; 276 (53.8%) agreed and strongly agreed on difficulty to understand online classes without the appropriate guidance of the teacher; 229 (44.7%) disagreed and strongly disagreed on frustration to use a computer at home; 221 (43.1%) agreed on no face to face interaction with a teacher in online classes while s/he is presenting lectures; 206 (40.2%) students disagreed and strongly disagreed on the difficulty to afford the cost of internet for an online class, and 211 (41.1%) agreed on the difficulty to acquire significant information through online class only (Table 2).

**Table 2:** Students' attitude towards online classes.

Statements	SD n (%)	D n (%)	N n (%)	A n (%)	SA n (%)
It is difficult for me to afford the cost of the internet for online classes.	42 (8.2%)	164 (32.0%)	210 (40.9%)	72 (14.0%)	25 (4.9%)
It is difficult to understand online classes without the appropriate guidance of the teacher.	21 (4.1%)	105 (20.5%)	111 (21.6%)	199 (38.8%)	77 (15.0%)
Slow computers and poor internet connections discourage me to participate in the online classes.	21 (4.1%)	58 (11.3%)	81 (15.8%)	199 (38.8%)	154 (30.0%)
It is very frustrating to use a computer at home.	47 (9.2%)	182 (35.5%)	155 (30.2%)	91 (17.7%)	38 (7.4%)
There are no interactions with other fellow students in online classes.	21 (4.1%)	111 (21.6%)	90 (17.5%)	204 (39.8%)	87 (17.0%)
There is no face to face interaction with a teacher in an online class while s/he is presenting lectures.	20 (3.9%)	64 (12.5%)	101 (19.7%)	221 (43.1%)	107 (20.9%)
Students' and teachers' interaction is weak in an online class.	15 (2.9%)	75 (14.6%)	91 (17.7%)	214 (41.7%)	118 (23.0%)
It is difficult to acquire significant information on related subjects through online classes only.	9 (1.8%)	79 (15.4%)	95 (18.5%)	211 (41.1%)	119 (23.2%)
It requires a lot of mental effort to continu- ously use a computer for online classes.	23 (4.5%)	149 (29.0%)	114 (22.2%)	158 (30.8%)	69 (13.5%)
It is annoying to communicate through electronic mails when submitting assignments.	19 (3.7%)	113 (22.0%)	105 (20.5%)	181 (35.3%)	95 (18.5%)
I prefer the traditional face-to-face mode of classroom teaching than online classes.	9 (1.8%)	41 (8.0%)	118 (23.0%)	183 (35.7%)	162 (31.6%)
The online class is often avoided as it promotes social isolation.	19 (3.7%)	157 (30.6%)	155 (30.2%)	138 (26.9%)	44 (8.6%)
It is easy to read from print learning materials instead of electronic mediums or the internet.	15 (2.9%)	70 (13.6%)	89 (17.3%)	180 (35.1%)	159 (31.0%)

* Strongly disagree (SD), Disagree (D), Neutral (N), Agree (A), Strongly agree (SA)

Almost three-fifth (61.5%) of PCL nursing students had a negative attitude towards online classes. Similarly, more than four-fifth of BNS, BSN, and MBBS students had a positive attitude towards online classes (Table 3).

**Table 3:** Student's attitude towards online class.

Attitude	PCL Nursing n (%)	BNS n (%)	BSN n (%)	MBBS n (%)
Negative	51 (61.4)	13 (17.1)	16 (12.5)	37 (16.4)
Positive	32 (38.6)	63 (82.9)	112 (87.5)	189 (83.6)
Total	83 (100)	76 (100)	128 (100)	226 (100)

## DISCUSSION

COVID-19 lockdown had greatly influenced the conventional learning method of academic institutions across the world. During this period, the college administrations decided to take online classes as an alternative way to resume education. As the online classes are going on, it becomes crucial to identify students' attitudes to reveal their perspectives.

In the present study, around 70% of students agreed on slow computers and poor internet connections discourage them from participating in online classes. Lack of access to fast, affordable, and reliable internet connections certainly hinders online learning for the students. The study conducted in Uganda among undergraduate medical and nursing students also identified that poor internet connectivity (84%) and costs (93%) were the most important barriers to e-learning.^7,12^ But in the present study, around 40% of students disagreed, and another 40% were neutral on affordability issues.

Around 55% of students from this study agreed and strongly agreed on difficulty understanding online classes without appropriate guidance by their teachers. This finding is in line with similar studies from Saudi Arabia's medical students, higher education students from Romania, and senior secondary students from India. They agreed that the educator's e-learning skills and teaching style to the online environment might affect the learning process. Similarly, technical issues were seen as most important, followed by teachers' lack of technical skills to teach online and support students learning online.^13-15^

Online learning can create learning flexibility without being limited by distance, space, and time. For effective online learning, the latest technology must be available to improve the learning process and interactions between teachers, students, and technicians.^16^ In the present study, 43.1% of students were agreed on no face to face interaction with a teacher in online class while presenting lectures, 41.7% agreed on weak interaction with students and teachers in an online class, and 39.8% of students agreed on no interaction with other students in an online class. The lack of face-to-face interaction with the instructor, response time, and absence of traditional classroom socialization were identified as online learning problems as highlighted by higher education students from Pakistan.^7^ In UAE also, 57.9% agreed that distance learning made group collaboration less and very limited. In the same study, most students agreed that due to the limitation of face-to-face interaction in the online learning system, it is sometimes difficult to get a proper explanation of some materials.^17^ In India, students, favored online learning to sustain their academic interest and development during this pandemic, yet, they perceived many challenges during online learning like lack of face-to-face interactions, lack of socialization, distraction by social media, and technology-related issues.^18^ From the perspective of undergraduate dental students from Indonesia, peer-to-peer communication and interaction in a group are not often feasible in the virtual learning method.^19^ Another study conducted among 83 undergraduate students in the Peshawar district also documented similar findings in these areas.^20^

COVID-19 had an unprecedented impact on medical education worldwide, leading to the cancellation of lectures, placements, exams, and electives.^21^ Learning activities previously done by the face-to-face method in the classroom switched to the online learning system. Both faculties and students are expected to remain at home and must carry out their duties and responsibilities to keep students informed of proper education and teaching.^22^ But more than two-thirds of students from the present study agreed and strongly agreed on preferring the traditional face-to-face mode of classroom teaching than online classes. In Libya also, only 38.2% of medical students agreed that e-learning could replace traditional teaching methods.^23^ In Pakistan, students, did not prefer e-teaching over face-to-face teaching during the lockdown situation and suggested that administration and faculty members take necessary measures to improve e-teaching for better learning.^24^ In India, more than half of the third-year MBBS undergraduate students, felt understanding concepts and retention of the topic was better in classroom learning.^18^ Similarly, more than three-fifth of undergraduate dental students from Indonesia disagreed that distance learning gave similar learning satisfaction to classroom learning. The survey demonstrated that only 44.2% of students preferred distance learning over the classroom.^19^ All these studies highlighted the student's preference for conventional learning than online learning.

In the present study, around 31% of students were agreed, and 13.5% strongly agreed on using a computer for class requires much mental effort. The study conducted among 100 senior secondary school learners from Delhi also documented that back-to-back classes were mentally exhausting for about 67.5% of the learners. The freedom and flexibility to study at one's own pace encouraged more than 40% to want to continue studying through online learning.^15^

In the present study, almost three-fifth (61.5%) of PCL nursing students had a negative attitude towards online class, and more than four-fifth of BNS, BSN, and MBBS students had a positive attitude towards it. In Uganda, an overall negative attitude towards e-learning was identified among medicine and nursing students, where the mean attitude scores were increased with an increase in internet connectivity quality.^12^ In Pakistani students, negative perception about e-learning had been identified by 77.4% students, out of which 86% students felt e-learning has little impact on their learning.^24^ But in India, the positive perception was identified among most students towards e-learning, and thus, acceptance of this new learning system is high.^25^

Study findings were based on students' self-reported data, and the quality depends on students' faithful responses.

## CONCLUSIONS

The study concludes that MBBS and bachelor-level nursing students from NAIHS have a positive attitude towards online classes, yet most PCL nursing students have a negative attitude towards it. Poor internet connection, less group collaboration, and technical difficulties often negatively affect online learning. Though online learning is seen as effective during the COVID-19 pandemic, students from NAIHS prefer traditional face-to-face learning. Therefore, faculties and academic institutions are encouraged to design more collaborative undergraduate courses to enhance learner-learner interaction and learner-teacher interaction. All these activities are essential to develop and maintain a positive attitude among students towards their online learning; thus, teaching-learning becomes more effective.
